# Role of T Lymphocytes in Glioma Immune Microenvironment: Two Sides of a Coin

**DOI:** 10.3390/biology13100846

**Published:** 2024-10-21

**Authors:** Laiba Noor, Arun Upadhyay, Vibhuti Joshi

**Affiliations:** 1 Department of Biotechnology, Bennett University, Greater Noida 201310, Uttar Pradesh, India; 2 Department of Bioscience and Biomedical Engineering, Indian Institute of Technology Bhilai, Durg 491002, Chhattisgarh, India

**Keywords:** glioma, tumor microenvironment, T lymphocytes, immunomodulation, immunotherapy

## Abstract

**Simple Summary:**

Gliomas are diverse types of tumors originating from glial cells. Among these tumors, glioblastoma multiforme (GBM) is the most aggressive form and has a poor prognosis and high mortality rate, with treatment success largely influenced by patient age, tumor location, and genetic mutations. Glioma cells can reprogram their tumor microenvironment (TME) to support tumor proliferation and survival, interacting with other cells through cytokines and growth factors. The TME of gliomas is highly heterogeneous, complex, and often immunosuppressive, interfering with treatment strategies. Current therapeutic strategies focus on modulating immune cell activities and enhancing anti-tumor responses. T-cell-based immunotherapies are being explored to target glioma cells. These include chimeric antigen receptor (CAR) T-cell therapy and T-cell receptor (TCR) therapy, which involve engineering T cells to recognize tumor antigens. However, these therapies face multiple challenges including tumor heterogeneity, immunosuppression by T cell exhaustion, and altered antigen presentation, etc. Future research should focus on boosting T cell functions, targeting immune checkpoints, and developing more effective delivery methods to improve treatment outcomes for glioma patients.

**Abstract:**

Glioma is known for its immunosuppressive microenvironment, which makes it challenging to target through immunotherapies. Immune cells like macrophages, microglia, myeloid-derived suppressor cells, and T lymphocytes are known to infiltrate the glioma tumor microenvironment and regulate immune response distinctively. Among the variety of immune cells, T lymphocytes have highly complex and multifaceted roles in the glioma immune landscape. T lymphocytes, which include CD4^+^ helper and CD8^+^ cytotoxic T cells, are known for their pivotal roles in anti-tumor responses. However, these cells may behave differently in the highly dynamic glioma microenvironment, for example, via an immune invasion mechanism enforced by tumor cells. Therefore, T lymphocytes play dual roles in glioma immunity, firstly by their anti-tumor responses, and secondly by exploiting gliomas to promote immune invasion. As an immunosuppression strategy, glioma induces T-cell exhaustion and suppression of effector T cells by regulatory T cells (Tregs) or by altering their signaling pathways. Further, the expression of immune checkpoint inhibitors on the glioma cell surface leads to T cell anergy and dysfunction. Overall, this dynamic interplay between T lymphocytes and glioma is crucial for designing more effective immunotherapies. The current review provides detailed knowledge on the roles of T lymphocytes in the glioma immune microenvironment and helps to explore novel therapeutic approaches to reinvigorate T lymphocytes.

## 1. Introduction

Glioma refers to a diverse class of primary central nervous system (CNS) tumors that originate from glial cells [1]. The glial cells constitute a major population of cells in the brain surrounding neurons. While neurons are responsible for functions like transmitting electrical impulses and chemical signals to and from the brain, glial cells act as glue and provide various types of support, including protection, cleaning, and nourishment to neurons [2]. Gliomas are a broad category of primary malignant tumors accounting for 80% of all CNS malignancies [3]. The World Health Organization (WHO) has categorized gliomas as grade II-IV based on their histology and molecular features; for example, Grades II, III, and IV refer to the low-grade, anaplastic, and glioblastoma stages, respectively [4]. Glioma is typified by a persistently high patient death rate and a dire prognosis that impairs quality of life, as well as survival [5]. It is believed that a hierarchy of cellular states in glioma contributes to the heterogeneity of the tumor [6]. Gliomas are broadly classified based on the type of glial cells from which they originate, for example, astrocytomas, ependymomas, and oligodendrogliomas. These tumors are further graded from I to IV based on their aggressiveness and growth [7,8]. Glioblastoma multiforme (GBM), a Grade IV condition, is the most notorious and malignant form, accounting for approximately sixty percent of all adult tumors [9]. Persistent headaches, vomiting, nausea, cognitive alterations, and seizures are some common symptoms of glioma, depending on the size and location of the tumor [1]. The use of surgical intervention, postoperative radiation, and subsequent chemotherapy creates a multi-modal treatment approach for aggressive tumors in the brain [10]. Higher-grade brain tumors have poor prognosis with a median survival rate of up to 15 months following diagnosis [11,12]. The success of treatment depends largely on location, genetic mutation, epigenetic dysregulation, and age of the patient. The complexity of these tumors, varied appearances and prognoses presents a great challenge for clinicians and scientists to understand biological behavior and design effective treatment strategies.

The tumor microenvironment (TME) is a highly heterogeneous system that consists not only of cancer cells, but also of many other brain resident cells, including a variety of immune-associated cell populations [13]. It is a complex system often influenced by dynamic changes in the composition of cell populations. Other factors contributing enormously to this heterogeneity are cell metabolites and chemical factors, like pH, oxygen, etc. [14,15]. Several reports indicate that glioma cells may reprogram their TME in such a way that it can support the proliferation, migration, and sustenance of tumor mass [16,17]. Glioma cells may interact with other cells via several cell surface molecules or by the secretion of cytokines, extracellular vesicles, and growth factors [18]. Notably, the glioma immune microenvironment is somehow distinct compared to many other tumors, including the presentation of a unique set of immune-modulating features that complicate treatment strategies several-fold. The glioma microenvironment is different from many other brain tumors primarily due to immunosuppression, dysfunctional T cells, unique cytokine and chemokine profile, and myeloid cell dominance [19,20]. The glioma immune microenvironment is characterized by the presence and involvement of various immune cells that interact in highly complex and often antagonistic ways. Remarkably, the complexity of the T lymphocyte-mediated immune responses creates multiple challenges for developing effective treatment [21,22,23]. However, immunotherapies aim to harness a patient’s immune system by directing immune cells to target and attack the tumor. More than 88 clinical studies investigating immunotherapies for glioma are now being conducted worldwide [24]. Novel immunotherapeutic strategies have been extensively studied in both preclinical and clinical settings, demonstrating significant responses. Clinical trials have shown promising efficacy with immune checkpoint inhibitors and CAR T cells. Additionally, exploring combinations of various immunotherapies, such as peptide vaccines, oncolytic virus vaccination, and immune checkpoint inhibitors, present exciting possibilities for future treatments [25,26]. Further research is needed to evaluate the potential of incorporating immunotherapy into the standard treatment regimen for glioma.

In this article, we are providing a detailed overview of how T lymphocytes promote immune evasion and play important roles in suppression of tumor cells. We will explore the highly dynamic interplay between T lymphocytes and the glioma microenvironment. This article provides an update to the literature on different experimental strategies being developed and tested by targeting T lymphocytes and explores possible therapeutic strategies.

## 2. Glioma Microenvironment: An Overview of Immune Landscape

There are several simultaneous processes involved in the origin and growth of glioma; for example, glioma cells’ engagement with multiple components of their surrounding environment plays a vital role in the sustenance of tumors [27,28]. The cells that make up the tumor microenvironment (TME) of gliomas are diverse and include vascular, immunological, and brain-resident cells [29]. A close interaction between all cellular components—malignant, endothelial, stromal, and immune cells—controls the homeostasis and survival of the TME [30]. These TME resident cells lie at the crossroads of gliomas’ temporal, spatial, and molecular heterogeneity.

**Figure 1:**
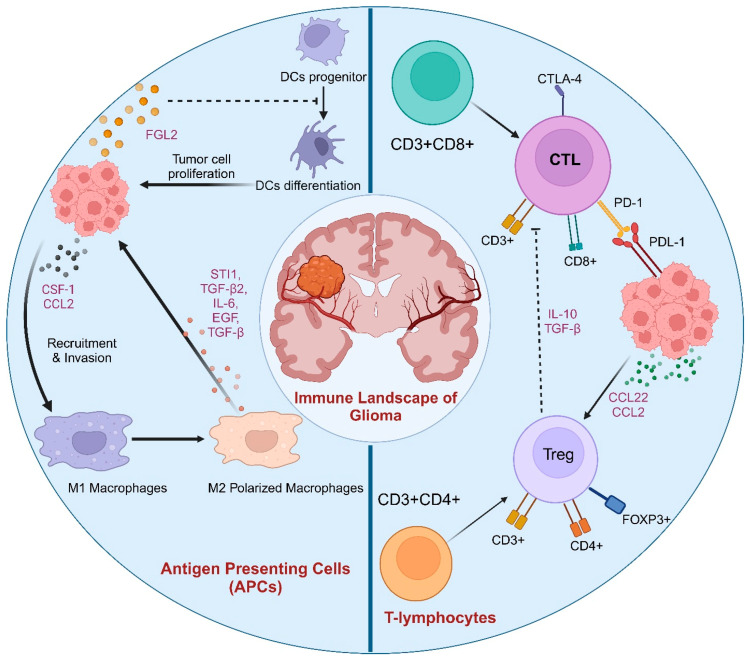
Immune landscape of glioma: The major cell population representing the glioma immune landscape consists of antigen-presenting cells, including M2 macrophages, whereas Treg and exhausted cytotoxic T cells are among the T lymphocytes present in higher numbers.

The diverse population of immune cells, including microglia, macrophages, T cells, B cells, and natural killer (NK) cells, creates a highly complex and immunosuppressive environment, as shown in Figure 1. Various additional factors further add to this complexity (See Table 1) [31]. A large number of tumor-associated macrophages (TAMs) are prevalent in the glioma microenvironment. TAMs may exhibit both pro- and anti-tumor roles [32,33]. This primarily depends on their polarization, which can further advance tumor growth by secreting multiple pro-angiogenic factors, such as vascular endothelial growth factor (VEGF), and suppress anti-tumor immune responses. Targeting macrophages is a promising strategy for glioma therapies. For example, targeting pathways such as the colony-stimulating factor 1 receptor (CSF-1R) could reduce macrophage infiltration and promote the activation of cytotoxic T cells [34,35]. Microglia, the principal immune cell population in the CNS, play crucial roles in the glioma microenvironment. Their polarization towards M2, a pro-tumor phenotype, may foster tumor growth and invasion by releasing cytokines. These cytokines may suppress anti-tumor immunity and promote angiogenesis [14,32]. Microglia may also contribute to tumor resistance towards various treatment strategies, thereby making a critical target for improvement in therapeutic responses. T cells are very crucial for adaptive immunity and can present anti-tumor effects in gliomas. Regulatory T cells (Tregs) and various immunosuppressive cytokines released by glioma cells may often suppress their functions. This leads to several challenges for T cells that provide an efficient response against gliomas [36,37]. T-cell dysfunction has different mechanisms, such as anergy, exhaustion, and tolerance. T-cell anergy represents the state of hyperresponsiveness when T cells encounter antigens but lack costimulatory signals. Glioma has reduced IL-2 production that enhances T cells’ anergy. Further, the glioma tumor environment generates T-cell tolerance by suppressing effector T-cell activation and responses toward tumor antigens. The major mechanism that impacts T cells’ function in glioma is exhaustion which includes an increase in the expression of inhibitory receptors like PD-1, TIM-3, and Lag-3 [38,39].

Another important immune cell population, B cells, promote angiogenesis and immune suppression by secreting pro-angiogenic factors and immunosuppressive cytokines. They also have significant effects on tumor durability and immune evasion. Modulating B cell activity could be a possible approach towards developing novel glioma treatment strategies [23,46]. Natural killer (NK) cells may pose toxic effects against glioma cells, and hence may contribute to controlling tumor progression, specifically via its interactions with major histocompatibility complex class I (MHC-I) components. NK cells induce apoptosis in target cells through contact-dependent cytotoxicity, utilizing granzyme B and perforin to initiate the process [47]. NK cells have also demonstrated the ability to control tumor growth by secreting cytokines, which are facilitated by the natural cytotoxicity receptor NKp44 [48]. However, these cells often get functionally impaired in the glioma tissues, either due to the presence of inhibitory ligands or the secretion of immunosuppressive cytokines. Several therapeutic strategies aim to rejuvenate NK cell activity and enhance its effects through combination treatments while overcoming immunosuppressive signals to effectively target tumor cell growth [49,50]. Chimeric antigen receptor and NK-cell-based (CAR-NK) therapies are being investigated in ongoing clinical studies. Zhang et al. evaluated the safety and tolerability of intracranial injections of NK-92/5.28.z CAR NK cells in patients with recurrent HER2-positive glioblastoma. The study aimed to determine the maximum tolerated dose for advancing to a phase 2 clinical trial (NCT03383978) [51]. Another clinical trial is enrolling patients with relapsed or refractory solid tumors, including malignant gliomas, to evaluate a novel CAR-NK-cell immunotherapy specifically targeting the MUC1 antigen (NCT02839954) [52]. An additional cell population, called myeloid-derived suppressor cells (MDSCs), are highly prevalent in a glioma microenvironment. Targeting MDSCs may lead to enhanced anti-tumor responses and diminished tumor progression in preclinical models [53,54].

The tumor microenvironment, comprising cellular and acellular components, plays a crucial role in gliomagenesis. Alterations within the TME have been demonstrated to drive glioma development, progression, invasion, and resistance to therapy [55,56]. Overall, as a component of the intricate microenvironment, tumor-infiltrating immune cells may be important in both supporting and inhibiting tumor development [57]. Considering the complexity of the diverse cell populations in the glioma TME, the development of more focused strategies exploiting the specific roles of distinct immune cells may hold high potential for advancing glioma treatment approaches. In upcoming subsections, we are discussing major immune cell populations for their involvement and vital roles in a glioma microenvironment.

### 2.1. Tumor-Associated Macrophages

TAMs are the primary immune components of gliomas, making up approximately 30–50% of the tumor mass. Glioma tissues contain a heterogeneous population of macrophages, for example, bone marrow-derived macrophages and tissue-resident microglia [58,59]. There are two separate origins for tumor-associated macrophages: microglia in the brain and monocytes from bone marrow. Embryonic yolk sac progenitor cells give rise to microglia and are not regenerated by peripheral mononuclear hematopoiesis after birth [60]. It is believed that local proliferation and extended cellular lifetime are responsible for microglia maintenance in the normal adult brain [60,61]. Likewise, non-parenchymal macrophages regulate immune responses at the brain’s boundaries [62]. In the CNS, they originate during the embryonic stage and maintain their population through self-renewal [63]. They are predominantly stable populations throughout adulthood [62]. In contrast, circulating monocytes are drawn to the brain parenchyma during disruptions to tissue homeostasis or pathological conditions, where they differentiate into bone marrow-derived macrophages (BMDMs). These cells are replenished through monocytosis, especially as the tumor progresses and increases susceptibility to inflammation.

In the CNS, microglia are the primary source of active immune surveillance. When inflammation starts, microglia can move quickly towards the lesion, where they engulf and clear debris, releasing cytotoxic molecules, including cytokines, proinflammatory, proteinases, and reactive oxygen intermediates. However, in glioma, the microglia are reprogrammed in such a way that their anticancer (cytotoxic) effects are weakened. Glioma cells exhibit increased migration and invasiveness in vitro in the presence of microglia [64]. The infusion of ganciclovir (a particular substrate for the viral thymidine kinase that acts as an apoptotic inducer and triggers a mitochondrial Bcl-2 death pathway) in CD11b-HSVTK mice significantly reduced glioma growth by depleting TAMs [64,65]. TAMs produce many proteins that can enhance glioma proliferation and/or migration, such as stress-inducible protein 1 (STI1), epidermal growth factor (EGF), colony-stimulating factor 1 (CSF-1), chemokine ligand 2 (CCL2), interleukin-6 (IL-6), and transforming growth factors: TGF-β and -β2 [58].

### 2.2. Dendritic Cells

The professional antigen-presenting cells (APC), the dendritic cells (DCs), are essential to both innate and adaptive immune responses. The two different subsets of DCs are called myeloid DCs (mDCs) and plasmacytoid DCs (pDCs). In a murine model, pDCs have been shown to promote glioma progression. Depleting pDCs in these mice enhances their survival by reducing both the number and the suppressive functions of Tregs [66]. Moreover, the normal functioning of DCs can be affected by TGFβ and IL-10 cytokines, released by glioma cells. In glioma, tumor cells secrete fibrinogen-like protein 2 (FGL2), which inhibits the development of granulocyte-macrophage colony-stimulating factor (GM-CSF)-induced CD103^+^ DCs. This is due to the suppression of the activation of nuclear factor kappa-B (NF-κB), signal transducer and activator of transcription (STAT1/5), and p38 in DCs. As a result, CD8^+^ T cells are neither primed nor activated, leading to the progression of glioma [42].

### 2.3. T Lymphocytes

The majority of lymphocytes are made up of CD4^+^ and CD8^+^ T cells. CD8^+^ T cells play a vital role in tumor clearance, and a higher presence of tumor-infiltrating CD4^+^ and CD8^+^ T cells is linked to extended patient survival in glioma [67]. During tumor evolution, brain cancer cells devise strategies to evade T-cell antitumor responses. Glioma cells suppress immune activation by secreting TGF-β and IL-10, while also reducing MHC class II expression on monocytes [43]. One of the most well-known lymphocyte-inhibiting regulators is PD-L1. A worse prognosis for individuals with gliomas is predicted by higher expression of PD-L1 [68]. Expression of PD-L1 in circulating TAMs and monocytes can be upregulated by glioma cells via autocrine/paracrine IL-10 signaling [69]. T-cell activation is negatively regulated by another immunological checkpoint protein called cytotoxic T-lymphocyte associated protein 4 (CTLA-4). Elevated expression of CTLA-4 is observed in samples of high-grade gliomas, and this is attributed to the absence of CD80/86 co-stimulatory molecule expression [70]. Factors like proliferating and dying tumor cell self-antigens can attract Tregs to tumor lesions. Notably, TGF-β produced by tumor cells and DCs can significantly enhance Treg enrichment. Glioma cell-secreted soluble molecules, such as (C-C) motif ligand 22 (CCL22) or (C-C) motif ligand 2 (CCL2), draw Tregs to the surrounding tumor *milieu*. An essential immune escape strategy for gliomas is the accumulation of Tregs [71,72]. Sayour et al. revealed that higher Tregs were linked to worse survival rates and early tumor recurrence in glioma patients [73].

## 3. Roles of T-Cells in Anti-Tumor Immunity

The human body is constantly monitored by APCs. When they come across tumor-specific antigens, they engulf and present those on the surface of MHC class II molecules. Afterward, when DCs reach the secondary lymph nodes, T cells and naive T cells, including cytotoxic T lymphocytes, identify MHC class II peptides [74]. Naive CD4^+^ T cells can differentiate into various T helper (Th) cell types, such as Th1, Th2, Th9, Th17, and regulatory T cells [75]. The CD4^+^ cells are classified as Th1 or Th2 cells based on the cytokines they produce. In the last several decades studies have demonstrated the important roles that CD4^+^ T lymphocytes play in rejecting human tumors. By stimulating cytotoxic CD8^+^ T cells, they can aid in CD8^+^-mediated tumor eradication. Furthermore, some CD4^+^ T cell subsets can actively aid in eliminating tumor cells [76]. Differentiated cytotoxic lymphocytes known as CD8^+^ T cells serve as effector molecules in the deactivation of CD4^+^ T cells. Nevertheless, they lack effector-memory function, may be less efficient in suppressing tumor cells, and are more vulnerable to tumor rejection fatigue, suggesting that CD4^+^ T cells retain CD8^+^ T cells’ efficacy and capacity to develop into effector memory CD8^+^ T cells by inducing the activation and maturation of these cells [77].

When CD4^+^ T lymphocytes encounter MHC class II molecules, they produce IFN-γ, which prompts host cells to eliminate tumor cells. IFN-γ stimulates the release of two CXC chemokines (C-X-C motif) ligands: interferon-gamma inducible protein 10 (IP-10) and migration-inducing gene (Mig). By disrupting the tumor vasculature, these chemokines show anti-angiogenic properties, resulting in growth suppression and tumor demise. Furthermore, IFN-γ stimulates macrophages to produce nitric oxide and TNF. However, in the absence of antigen-stimulated CD4^+^ T cells within the TME, IFN-γ alone is insufficient. The combined activity of CD4^+^ T cells and IFN-γ is essential for activating TNF, IP-10, Mig, and other tumoricidal cells [76]. According to research conducted on melanoma patients, mutant neoantigens are often recognized by CD4^+^ T lymphocytes [78]. Additionally, in three mouse tumor models, CD4^+^ T cells identified the immunogenic mutanome of non-synonymous cancer mutations more often than CD8^+^ T cells [79]. Moreover, administering CD4^+^ cytotoxic T cells can trigger an anti-tumor response. In bladder tumors, clonal expansion of cytotoxic CD4^+^ subsets are probably due to tumor antigen recognition. These CD4^+^ subsets can destroy autologous tumor cells ex vivo due to their secretion of TNF-α and IFN-γ [80]. This shows other tumors support T cells for their anti-tumor functions. However, this is not the case with glioma. In the next section, we will discuss how T cells play their role in the glioma microenvironment.

## 4. How Do T Lymphocytes Influence Glioma Microenvironment?

**Figure 2:**
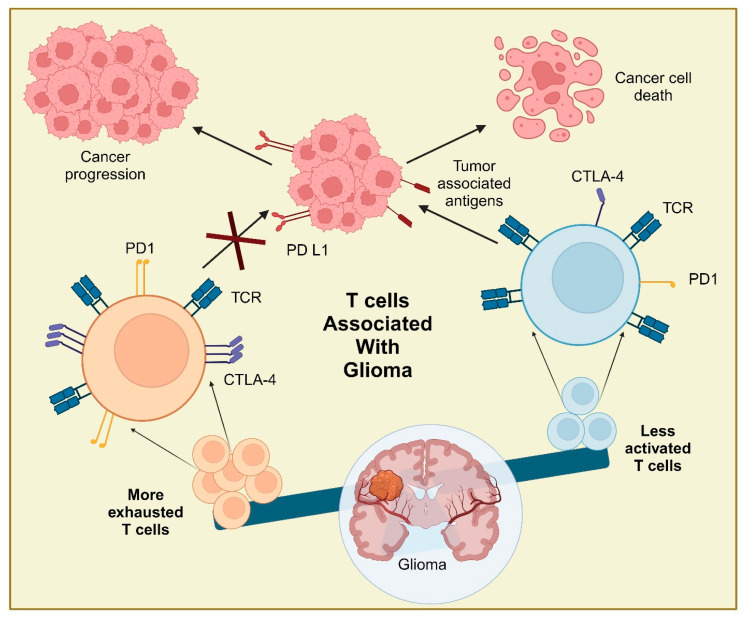
Glioma-associated T cell: The immunosuppressive effect of the glioma tumor microenvironment is the result of an increase in tumor-promoting terminally exhausted T cells and fewer tumor antigen-recognizing cytotoxic T cells.

T cells are part of the adaptive immune system and keep circulating in the blood to identify and eliminate pathogens. However, the blood–brain barrier (BBB) restricts entry of circulating T cells into the brain. While the BBB is essential to maintaining neuronal homeostasis, it also poses the challenge of immune surveillance in the brain. Although the BBB may get disrupted in glioma, it still limits infiltration of circulating T cells. Only a few T cells are found in the parenchyma and perivascular space in the adult healthy brain [81,82]. T cells keep carrying the immune surveillance of the brain through cerebrospinal fluid [83]. Many brain pathologies affect the integrity of the BBB and damage the tight junctions, which lead to leakage and entry of immune cells into the brain or CNS [84,85]. T cells rarely enter the CNS through the BBB; rather, they enter through the more permeable blood–cerebrospinal fluid barrier (BCSFB). Choroid plexuses express many adhesion molecules that help activated T cells cross the BCSFB [86,87]. Meningeal venules also allow the entry of activated T cells to some extent [87]. However, a major population of T cells only enters when neuroinflammation changes the permeability of the BBB and other barriers [88]. Brain tumors are also known to damage physical barriers of the brain, including the BBB and brain-cerebrospinal fluid barrier (BCSFB), and make new, more permeable brain tumor barriers (BTB) [89,90]. This further results in the slow movement of lymphocytes into the brain with the help of endothelial cells and an increase in brain antigen expression [91]. Decreases in junctional proteins and aberrant distribution of pericytes and astrocytes end feet loss make the BTB more permeable than the BBB and facilitate entry of immune cells [92]. Majorly, CD8^+^ T cells, due to the presence of chemokines, macrophages, and CD4^+^ T cells, are permissible to cross the BTB [93,94]. There are multiple different types of T lymphocytes that circulate in blood [95]. However, brain tumor-infiltrating lymphocytes (TILs) are very limited in type and number. The major types of TILs found in glioma patients are CD3^+^, CD3^+^/CD8^+^, CD3^+^/CD4^+^, CD3^+^/CD4^−^CD^−^, and CD3^+^/CD4^+^ CD8^+^ as observed in patient-derived tumor cell lines [96]. The amount of T cell subsets, like CD8^+^ T cells, also depends on the grade of the tumor [97]. Moreover, multiple studies have reported a significant presence of CD4^+^ CD25^+^FoxP3^+^ T regulatory cells in the glioma microenvironment [98]. Another subset of T cells, NKT cells, are also under investigation for their presence in gliomas, as their antigen-presenting molecule CD1d expression has been found in glioma patients [99]. Similarly, ligands like natural killer group 2, member D (NKG2D), found in glioma that are recognized by gamma delta (γδ) T cells, enhance the possibility of their infiltration into a glioma [100,101]. In summary, the immunosuppressive environment of a glioma majorly supports exhausted CD8^+^ and Treg cells, (please see Figure 2) [40]. This also suggests the need to further explore the glioma TME with more advanced methods and a deeper understanding of T cell subsets with glioma.

## 5. T Lymphocytes: Cells with Dual Roles in Brain Tumors

T cells are essential for the adaptive immune response against tumors. T lymphocytes can act as both promoters and inhibitors of glioma growth. Pro-tumoral T cells, like Tregs promote the growth of tumors, while anti-tumoral T cells like CTLs eradicate the tumor cells [102]. Although CD8^+^, Treg, and conventional CD4^+^ T cells make up 1–5% of glioma cells, most of them lack anti-tumor activity [103]. Infiltration of CD8^+^ T cells in newly diagnosed glioblastoma patients reported prolonged survival. It has been reported by Han, S et al., that higher grade tumors have fewer CD8^+^ T cells as come to lower grade tumors [104]. However, the level of infiltration and its correlation with improved outcomes varies among studies. Mauldin IS et al. studied the IDH wild-type glioma patient samples and reported a correlation between improved survival and high density of proliferating CD8^+^ cells and also a high ratio of CD8^+^ to CD4^+^ cells [105]. Further, Xue C et al. also reported a positive association between overall survival and level of CD8^+^ T cell tumor infiltration [106]. Many other studies supported it by demonstrating the association of patient survival with infiltration of effector T cells [107,108]. However, the glioma tumor environment supports more Treg cells than CD8^+^ cytotoxic T cells. Majorly the mechanisms that lead CTLs to suppress the glioma TME include T cell tolerance, ignorance, energy, and exhaustion [38]. The only way to make these cells effective against cancer is the reversal of exhaustion [109]. Despite the inactivation of cytotoxic T-cells in glioma, they are the major targets for most of the current therapies and are continuously under exploration for new therapeutic efforts. A distinct subpopulation of T cells, Tregs, play a crucial role in maintaining immune homeostasis by exerting immunosuppressive effects. Treg cells have the ability to inhibit effector T-cell activation via several mechanisms, primarily the downregulation of co-stimulatory molecules on APCs and the release of immunosuppressive cytokines [110]. The glioma TME promotes Treg cells’ recruitment and survival by sustaining elevated cytokine levels that enhance Treg persistence [111]. Treg cells typically make up 5–10% of circulating CD4^+^ T cells, they are present in high numbers and frequencies are elevated in a variety of malignancies, where high Treg counts are linked to a poorer prognosis [112,113]. Glioma patients exhibit a higher proportion of circulating Tregs compared to healthy controls. Additionally, these patients show an increased infiltration of Tregs within the tumors [114,115].

Tregs express the molecular marker Forkhead Box P3 (FOXP3) transcription factor that plays a vital role in immunosuppression [116]. FOXP3 can inhibit the NFκB and Nuclear factor of activated T-cells (NFAT) signaling pathways, leading to reduced expression of key effector cytokines like IL-2 [117]. Treg cells are thought to be attracted to the TME through cytokines, such as CCL5-CCR5 and CXCL9/10/11-CXCR3 [118]. These cytokines are produced by the immune cells in the CNS and glioma cells. Tregs are exposed to favorable circumstances inside the tumor microenvironment, which facilitates their growth and survival and aids in the conversion of other T cells into Tregs through cytokines, including TGF-β and tumor-derived IL-10. Additionally, Tregs may further secrete IL-10 and TGF-β to enhance immunosuppression [119,120]. These molecules can suppress the activity of NK cells, and contribute to the development of MDSCs, and hinder the antigen-presenting function of dendritic cells [121,122]. T cell activation is a highly controlled process that is influenced by the antigen’s characteristics, the surrounding environment, and the length of time the T cell is exposed to the antigen. The subsequent T cell states may vary from an effector T cell state to a hyporesponsive T cell. The process usually starts when naive CD8^+^ T cells deliver a foreign antigen to APCs, which prompts their clonal expansion and differentiation into cytotoxic T cells. Following their expansion, these antigen-specific T cells lyse the cells that express that specific antigen [123]. It is important to recognize the tumor cells that disrupt the T-cell activation by hindering the antigen presentation, T-cell priming, or presentation, leading to the dysfunctional T-cell states that promote tumor growth. T-cell dysfunction within the glioma immune microenvironment manifests in various forms, including tolerance, ignorance, anergy, and exhaustion [44].

A primary way tumor cells evade immune detection is through the mechanism of T-cell tolerance. When T cells do not react to an antigen, they develop tolerance, which can be induced in the bloodstream or directly within the tumor. Tolerance can be attained through eliminating T cells that are reactive to antigens or by preventing T cells from activating after being exposed to antigens. The failure of T cells to respond to antigens can occur due to either the absence of positive co-stimulatory signals or the presence of negative co-stimulatory signals [44]. In gliomas, the expansion of Tregs can potentially induce tolerance by diminishing the activation signals from CD4^+^ helper T-cells to CD8^+^ T-cells [124]. T-cell ignorance refers to a condition where functional T-cells do not mount an effective immune response due to insufficient antigen exposure, distinguishing it from T-cell anergy [125]. In gliomas, this phenomenon occurs because tumor-associated antigens are often hidden from T cell detection, either through reduced tumor-associated antigens expression or physical barriers like the brain or tumor center. An immune-suppressive environment that lowers MHC expression by APCs can further impede antigen presentation in gliomas. Additionally, mature T cells in glioblastoma patients can be sequestered in the bone marrow of GBM patients or mice, preventing them from effectively responding to the tumor [126,127]. APCs in gliomas express less of the costimulatory ligands CD80 and CD86, which inhibits T-cell activation. This deficit leads to T cell anergy and insufficient stimulation of tumor-specific T lymphocytes [128]. It is crucial to differentiate between “adaptive tolerance” and “clonal anergy”. While adaptive tolerance results from persistent low-level antigen exposure, reduced zeta-chain-associated protein kinase 70 (Zap70) activity, and limited calcium-induced NFκB signaling. Clonal anergy is caused by defective co-stimulation and altered Ras/mitogen-activated protein kinase (RAS/MAPK) signaling pathways [129,130].

T cell anergy and T cell exhaustion share a variety of phenotypic and epigenetic characteristics. T cell exhaustion, as opposed to T cell anergy, is caused by naive T cells being continuously stimulated by antigens, which leaves them in a hypofunctional condition [127]. Transcription factors, like T-box expressed in T cells (T-bet), NFAT, and Eomesodermin (Eomes) are involved in programmed T-cell exhaustion. T-cells that are exhausted have reduced T-bet expression and elevated Eomes expression [131]. In the exhausted state, inhibitory immunological checkpoints like PD-1 and CTLA-4 are expressed leading to NFAT’s inability to bind to AP-1 [132]. Several newly identified checkpoints that play a role in T-cell exhaustion include T cell immunoglobulin and mucin-domain containing-3 (TIM-3), CD39, B and T lymphocyte attenuator (BTLA), T cell immunoreceptor with Ig and ITIM domains (TIGIT), lymphocyte activating 3 (LAG-3) [133]. In glioma, TILs were severely hypofunctional due to the co-expression of PD-1, TIM-3, and LAG-3 which will prevent from lysing tumor cells through cellular programming [133,134].

## 6. T Cell-Based Approaches of Immunotherapy for Glioma

In addition to these, multiple T cell based immunotherapeutic approaches have been investigated to target glioma cells. The most worked out method is chimeric antigen receptor (CAR) T-cell therapy, in which a patient’s T cells are genetically modified to express CARs that can target tumor antigens associated with glioma. The modified CAR T cells can effectively recognize and target glioma cells. However, multiple challenges in the effectiveness of this therapy have been reported, for example, the immunosuppressive glioma microenvironment, heterogeneous antigen expression in tumor cells, and insufficient trafficking to the site of the tumor [135,136]. Similarly, in dual-target CAR T-cell therapy, T cells are engineered to target two different antigens together to enhance their efficacy [137]. Another approach is T-cell receptor (TCR) therapy that involves T-cell engineering to express those receptors that can recognize specific peptide–MHC complexes derived from tumor-associated antigens. This approach enhances the ability of T cells to recognize and destroy glioma cells [138,139]. Initial trials have shown promising results in reducing tumor size, suggesting potential improvements in treatment outcomes compared to single-target therapies.

Although emerging T-cell-based therapies for glioblastoma show promising results in early clinical trials, several challenges remain. The multi-epitope mRNA vaccine CVGBM, which targets tumor-associated antigens on HLA-A02:01 and HLA class II molecules, has progressed to a Phase I trial (NCT05938387) in newly diagnosed, resected, Methylguanine–DNA methyltransferase (MGMT)-unmethylated glioblastoma patients. Pre-clinical studies indicated strong CD8^+^ and CD4^+^ T-cell responses, and the trial with 16 participants showed a tolerable safety profile, leading to further evaluation of dose. Another study (NCT03233152) combining nivolumab, ipilimumab, and myeloid dendritic cells for recurrent high-grade gliomas demonstrated a median progression-free survival of 24 weeks and a 50% 1-year overall survival rate. Additionally, a Phase I trial (NCT06018363) of QH104 CAR-γδT cells in recurrent glioblastoma reported a 42.9% objective response rate and a 100% disease control rate without severe side effects. These advances underscore the potential for overcoming glioma’s immunosuppressive microenvironment and antigen heterogeneity, which are key challenges for T-cell therapies.

Like CAR T-cell therapy, TCR therapy faces obstacles due to complexities associated with antigen presentation and immunosuppression in glioma [140]. The immunosuppressive effect is primarily mounted by population of MDSCs and regulatory T cells that inhibit effector T cells. MDSCs strengthen this by promoting secretion of inhibitory cytokines and expressing inhibitory receptor proteins. Production of reactive oxygen species, secretion of nitric oxide, expression of IL-10, TGF-β, and arginase are other possible factors contributing to T cell exhaustion; therefore, immune suppression in glioma microenvironment and poses a challenge for various therapeutic strategies [39,43,141]. Another factor that challenges T cell-mediated therapies is the heterogeneity of tumor cells. The highly variable tumor population may have cells that do not express antigens recognizable by modified/engineered T cells. More innovation and inclusion of new therapeutic strategies may be helpful in addressing the above challenges. Combination therapy including radiation of gene editing for improvising T cells effectiveness may provide more success in the future.

A new line of therapeutics combines T-cell therapies with immune checkpoint inhibitors, e.g., anti-PD-1 or anti-CTLA-4 [142,143]. This approach enhances immune response against gliomas by addressing immunosuppressive features of the tumor microenvironment, and hence increases the effectiveness of T-cell therapies. Multiple efforts have also been made to target T cell exhaustion [144]. One of the case studies targeted reduction in IL-10 signaling, a major factor of T cell exhaustion in glioma microenvironment, by Janus kinase/signal transducer and activator of transcription (JAK-STAT) inhibitor, Ruxolitinib; however the patient showed stabilization for 8.5 months and was still alive up to the reporting of results [145]. Another pre-clinical study reported biomimetic nanoparticles loaded with paroxetine (PX) reduced T cell exhaustion by decreasing the expression of PD1 and TIM 3 on T cells and enhancing the survival of glioblastoma-bearing mice [146]. These efforts are made to overcome the challenges of the immunosuppressive environment of glioma. However, the success rate is low and needs more optimization for future clinical applications. One major obstacle in the treatment of gliomas or brain tumors is the BBB. Increasing T cell trafficking across the BBB is one of the biggest challenges in glioblastoma targeting. Chemokine-modulation-increased IL-1 signaling and blocking of TGF-β signaling are some of the efforts aimed at increasing T cell infiltration across the BBB [143,147]. To address this, several additional treatment methods are included in the glioma therapy plan. For example, intrathecal infusion of T cells increases the accessibility and efficiency of T-cell therapies [148,149]. This strategy addresses some of the limitations of systemic administration and potentially enhances T cell interactions with glioma cells. Glioma stem cells (GSCs) are believed to drive tumor recurrence and treatment resistance [150,151]. A recent advancement in glioma therapy focuses on designs to improve long-term outcomes for patients with gliomas. Overall, the T cell-based immunotherapy approaches involve multiple methods that may potentially address the challenges associated with tumor microenvironments, antigen heterogeneity, and the blood–brain barrier.

## 7. Conclusions and Future Prospects

Being immune privileged, gliomas have been less studied for their immune microenvironment. The microenvironment and immune system interactions highlight the complexity and the challenges they pose for treatment of glioma. The immunosuppressive microenvironment of glioma complicates the effectiveness of immunotherapies. T lymphocytes, CD8^+^ cytotoxic T cells, and CD4^+^ helper cells play crucial roles in other tumors; however, in glioma immunity, they act as both anti-tumor agents and facilitators of immune evasion. T cell exhaustion and dysfunction, which often occur through the expression of immune checkpoint inhibitors, make a unique tumor microenvironment in gliomas, which further complicates therapeutic strategies. Advancements in T-cell engineering strategies and identifying suitable tumor antigens that can be targeted more effectively are possible future interventions in this direction. Developing successful combinations of T cell-based therapies with checkpoint inhibitors or immunomodulatory agents may overcome several challenges associated with current glioma therapy. Detailed investigations, including clinical trials of these methodologies, may provide substantial evidence on the efficacy of the proposed methods. For example, in a recent study, IL13Rα2-mediated CAR T cells containing CD28 transmembrane domain improved the efficacy of treatment [152]. Another phase I clinical trial delivering bivalent (targeting EGFR and IL13Rα2) CAR T cells to six patients intrathecally showed efficacy with preliminary safety against recurrent glioblastoma [137]. In recent years, there has been growing interest in using CRISPR/Cas9 gene-editing technology as a potential treatment for gliomas. This innovative technique holds great promise as a powerful tool for advancing glioma research, particularly in the development of targeted immunotherapies and novel drug therapies [153]. Emerging studies indicate that CRISPR/Cas9 can effectively induce autophagy in tumor cells, accelerate apoptosis, and potentially lead to the permanent eradication of tumor cells [154,155]. Another recently completed phase I trial on 65 patients with recurrent GBM utilized IL13Rα2 which targeted CAR T cells’ loco-regional administration showed promising results in a group of patients [156]. Engineering CAR T cells to target EGFR variant III leads to the production and secretion of T-cell-engaging antibody molecules and exhibits remarkable radiographic responses in multiple patients with recurring GBM [157].

**Figure 3:**
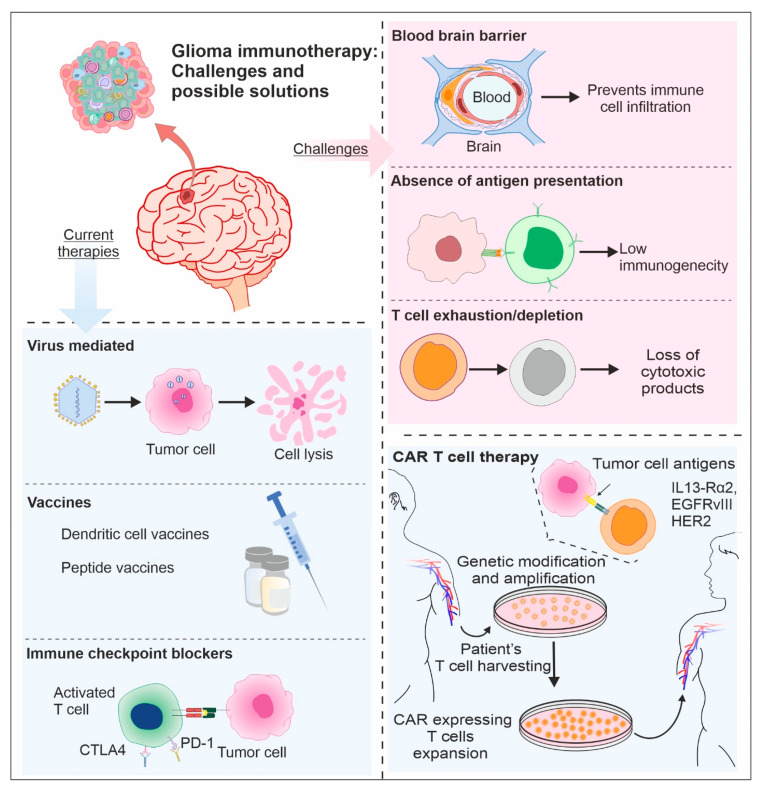
Glioma immunotherapy challenges and possible solutions: Current glioma immunotherapies are facing multiple challenges, primarily because of the blood–brain barrier, less antigen presentation, and T cell exhaustion. Future efforts in viral-mediated therapy, vaccines, and immune checkpoint blockade therapy are much needed, especially for glioma.

As discussed in previous sections, several other treatment strategies not related to CAR T cells have also been proposed and developed by various groups. Similar to T cell-mediated therapies, other methods may also have roadblocks. Despite several challenges, research is ongoing to overcome the BBB, T cell exhaustion, and suppressed antigen presentation in an effort to design effective therapeutic strategies against glioma. As presented in Figure 3, multiple immunotherapy approaches have been utilized in the past with varying success. For example, a combination of oncolytic viruses, such as Newcastle virus, vericular stomatitis virus, or vaccinia virus, with inducers of cell cycle arrest and apoptosis in temozolomide-treated glioma cells increased survival in the rat tumor model [158]. Similarly, the combination of DC vaccinations with radiation, chemotherapy, or both can cause DNA damage and endoplasmic reticulum stress, which in turn can trigger cell death. It can increase MHC and co-stimulator expression [159]. Furthermore, it fosters the release of chemokines and cytokines and decreases M2 macrophage activation, and therefore improves median survival rate and the body’s immune response [160]. Peptide vaccines are commonly employed to treat glioma targets like EGFRvIII (NCT01967758), and survivin (NCT01250470). Phase I peptide vaccination clinical trials are completed [161].

Another therapeutic approach utilizes immune checkpoint inhibitors. Cytotoxic T-lymphocyte antigen 4 (CTLA-4), programmed cell death 1 (PD-1), and programmed cell death 1 ligand (PD-L1) are the most often employed targets for checkpoint inhibitors [162]. In one of the studies involving such inhibitors, the effectiveness of anti-PD-1 nivolumab in comparison to anti-VEGF monoclonal antibody bevacizumab was investigated in patients with first-time GBM recurrence [163]. In another study, patients with newly diagnosed MGMT unmethylated glioma were tested for the effectiveness of nivolumab with RT vs. standard chemoradiotherapy [164]. Furthermore, another study assessed the effectiveness of nivolumab when combined with RT and TMZ, the standard of treatment, for patients with newly diagnosed MGMT methylated or indeterminate glioma [165]. In addition to all these approaches, as described in above sections, the CAR-T therapy has recently reported notable success. In summary, it is important to understand the dynamic interplay between T lymphocytes and glioma cells for developing effective immunotherapies. Future research should focus on exploring strategies like enhancing T-cell functionality, targeting immune checkpoints, and developing innovative delivery methods. Hopefully, by addressing these areas, future research can pave the way for more effective treatments for gliomas, ultimately improving patients’ survival rates and quality of life.

## Figures and Tables

**Table 1:** Key immunosuppressive mechanisms, their targets, and effects in Glioma microenvironment.

Modification	Mechanism	Targets and Effects	References
Recruitment of cells	Gliomas attract MDSCs, Tregs, and macrophages, creating immunosuppressive environment.	Increased production of STAT3	[39,40]
Altering functions of dendritic cells	TME cells render DCs dysfunctional.	Promote T cell exhaustion.	[41]
Secretion of cytokines	Gliomas secrete TGFβ-2, PGE, IL-1, IL-10 and FGL2.	Suppress the activity of effector cells	[42,43]
Immunosuppressive factors on surface	Glioma cells express PD-L1 that binds PD1.	Suppresses T cell activation, T cell anergy, apoptosis	[44]
Impaired antigen presentation	Glioma cells have altered MHC expression.	Hinders the identification of tumor antigens by T cells.	[45]

## Data Availability

No new data were created in this study.
